# Evaluation of the accuracy of primary CD4 gating in Senegalese individuals coinfected with HIV and tuberculosis

**DOI:** 10.1186/1742-4690-9-S1-P18

**Published:** 2012-05-25

**Authors:** Abdoul Aziz Diallo, Aliou Niang, Géraldine Daneau, Joséphine Khady Badiane, Makhtar Camara, Abdoul Almamy Hane, Luc Kestens, Souleymane Mboup, Tandakha Dieye

**Affiliations:** 1 Laboratory of Bacteriology, Dakar, Senegal

## Background

The classic multiparameter measurement of CD4+ T cells (standard method) by flow cytometry is complicated and expensive for resource-limited countries,. Simpler and less expensive methods like primary CD4 gating have been described but must be evaluated in patients where an overestimation of CD4 can be measured due to monocytes. To address this issue, we compared primary CD4 gating with the standard method in individuals coinfected with HIV and tuberculosis where the risk of CD4 T cell overestimation exists.

## Methods

Ninety eight patients were recruited, including 32 individuals infected with HIV alone (control group) and 66 individuals coinfected. Each fresh blood sample was analyzed within 6 hours with the fACSCalibur cytometer (Becton Dickinson) in the laboratory of Bacteriology-Virology of Le Dantec hospital in Dakar, Senegal. For each sample, 2 Trucount tubes were used: one for the standard method containing anti-CD3 FITC, anti-CD4PE and anti-CD45 PerCP monoclonal antibodies, and the other tube for the primary CD4 gating method containing only the anti-CD4 PE. Linear regression and Bland-Altman tests were used for statistical analysis.

## Results

The correlation of absolute CD4 T cell counts obtained by primary CD4 gating and the standard method was high for the HIV control and HIV-TB coinfected groups (R2 = 0.9897 and 0.9795, respectively). The mean bias was 15 cells/µl for the control group and 16 cells/µl for the coinfected group. For the interval < 200 cells/µl, the correlation is R2 = 0.9978 and 0.8327 (mean bias for both less than 7 cells/µl); for 200 - 500 cells/µl, the correlation was R2 = 0.8112 and 0.9624 (mean bias for both less than 2 cells/µl); for > 500 cells/µl, the correlation was R2 = 0.9809 and 0.9841, (mean bias for both less than 72 cells/µl).

## Conclusions

Primary CD4 gating is an affordable and accurate method for CD4 counting. With one antibody, it has the potential to be an effective alternative to complex panels for resource-limited countries. However, because of the intensive manual data analysis required, a highly skilled operator is necessary.

